# Machine Learning–Based Prediction Model Construction for Type 2 Diabetes Mellitus: A Comparison of Algorithms and Multilevel Risk Factor Analysis

**DOI:** 10.1155/jdr/4525736

**Published:** 2026-04-04

**Authors:** Qian Xu, Ruicong Yu, Huixin Qiu, Yannan Jiang, Jake Ball, Cuirong Xu, Jing Sun

**Affiliations:** ^1^ Operating Room, Zhongda Hospital Southeast University, Nanjing, Jiangsu Province, China, cis.seu.edu.cn; ^2^ School of Medicine, Southeast University, Nanjing, Jiangsu Province, China, seu.edu.bd; ^3^ Data Science Institute, University of Technology Sydney, Sydney, New South Wales, Australia, uts.edu.au; ^4^ Department of Nursing, Zhongda Hospital Southeast University, Nanjing, Jiangsu Province, China, cis.seu.edu.cn; ^5^ Heart Research Institute, University of Sydney, Sydney, New South Wales, Australia, sydney.edu.au

**Keywords:** health ecology, machine learning, prediction model, Type 2 diabetes mellitus

## Abstract

**Background:**

Against the backdrop of the global high incidence of Type 2 diabetes mellitus (T2DM), existing prediction models are largely confined to single‐dimensional risk factors, suffering from a core limitation of lacking multilevel integrated analysis. Given the severe impact of T2DM on individual health and healthcare systems, the construction of a comprehensive and accurate prediction model is of great significance.

**Objective:**

This study is aimed at constructing a T2DM prediction model, identifying multilevel risk factors, and enabling early screening, so as to help clinicians identify high‐risk individuals and provide targets for public health interventions.

**Methods:**

Data from the National Health and Nutrition Examination Survey (NHANES) 2021–2023 were used, including 6337 participants aged 18 years and older. Missing values were handled using Monte Carlo multiple imputation, collinearity was reduced via principal component analysis (PCA), and feature selection was performed using random forest (RF) and recursive feature elimination (RFE). The adaptive synthetic sampling (ADASYN) method was applied to address class imbalance. The performance of seven machine learning models, including decision tree, random forest, extreme gradient boosting (XGBoost), and adaptive boosting (AdaBoost), was compared.

**Results:**

The AdaBoost model exhibited the optimal performance, with an area under the curve (AUC) of 0.85 (95% confidence interval: 0.85–0.86), an accuracy of 0.71 (95% confidence interval: 0.70–0.72), and an *F*1 score of 0.71; its performance was further improved after parameter optimization. A total of 24 key risk factors were identified, including 19 at the individual trait level, 3 at the individual behavior level, and 2 related to working and living conditions.

**Conclusions:**

Machine learning models integrating multidimensional risk factors based on the health ecology framework can more accurately predict T2DM risk, providing a scientific basis for multilevel interventions. The innovation of this study lies in the first integration of the health ecology model with machine learning technology to systematically identify cross‐level risk factors. Compared with traditional models, it is more comprehensive, breaks through the limitations of previous studies, and provides a new and effective tool for the precise prevention of T2DM and public health interventions.

## 1. Introduction

Type 2 diabetes mellitus (T2DM) is a group of metabolic disorders characterized by hyperglycemia in the absence of treatment, accounting for 90% of all diabetes cases and posing substantial mortality and morbidity risks. As of 2021, the global prevalence of T2DM among adults aged 20–79 years is approximately 10.5%, affecting an estimated 536.6 million individuals [1], and this figure is projected to exceed 1.31 billion by 2050 [2]. Not only does T2DM directly cause around 1.5 million deaths annually, but it also induces a variety of severe complications such as blindness, renal failure, and cardiovascular diseases [3–7]. The global medical expenditures related to T2DM reached $966 billion in 2021 and are expected to rise to $1054 billion by 2045 [8]. Therefore, it is imperative to conduct an in‐depth exploration of its prevention and control strategies.

T2DM does not arise from a single causal factor but rather emerges as a product of complex interactions among multidimensional risk factors spanning individual physiology, lifestyle, socioeconomic status, and environmental exposures [9–13]. Ranging from microlevel factors such as insulin resistance and obesity, through meso‐level determinants including dietary patterns and physical activity habits, to macrolevel influences like educational attainment and environmental pollutant exposure, these elements interconnect and exert hierarchical impacts, collectively forming an intricate network underlying disease development.

This characteristic aligns closely with the core tenets of the health ecology model (HEM), a theoretical framework that emphasizes chronic diseases result from dynamic interactions across micro (individual), meso (community), and macro (social–environmental) systems [14, 15]. Only through comprehensive integration of factors at all these levels can we accurately elucidate disease pathogenesis and formulate effective prevention and control strategies [16].

However, most existing T2DM prediction models fail to follow this integrated logic and have significant limitations: Most models only focus on physiological indicators, failing to systematically integrate socioeconomic, behavioral, and environmental factors, resulting in single‐dimensional prediction and insufficient comprehensiveness; while some studies attempt to introduce multiple factors, they lack a health ecological model framework and consideration of hierarchical relationships and interaction effects between factors [17–20]. Traditional regression models have inherent limitations when dealing with high‐dimensional, nonlinear, multilevel data, making it difficult to capture complex variable relationships [21]. In contrast, ensemble machine learning models such as AdaBoost and extreme gradient boosting (XGBoost) are more adaptable as they can effectively mine potential patterns in high‐dimensional data, precisely handle nonlinear relationships and interaction effects between variables, and significantly outperform traditional methods in predicting accuracy in complex risk factor scenarios [22, 23].

Despite previous attempts to apply machine learning to T2DM prediction [18, 19], the aforementioned limitations have resulted in models with insufficient generalization ability and accuracy to meet the requirements of multilevel prevention and control. Therefore, this study is the first to integrate the HEM with seven machine learning algorithms. By employing RF/RFE for feature selection and ADASYN for data balancing, we systematically identify cross‐level risk factors to construct a more comprehensive and accurate T2DM prediction model. This approach provides a scientific basis for clinical screening of high‐risk populations and public health interventions.

## 2. Methods

### 2.1. Data Source

The data for this study were obtained from the National Health and Nutrition Examination Survey (NHANES), which is conducted by the National Center for Health Statistics (NCHS), a division of the Centers for Disease Control and Prevention (CDC). The survey is aimed at collecting health, nutritional, biochemical, and behavioral data from the noninstitutionalized civilian population of the United States through cross‐sectional surveys. Its sample design employs a multistage stratified probability sampling method, covering populations of different ages, ethnicities, and socioeconomic backgrounds, and is nationally representative. This study selected data from the 2021–2023 survey cycle, which included 11,933 participants. Data collection included household questionnaires (e.g., demographic characteristics, medical history, and lifestyle), physical examinations (e.g., height, weight, and blood pressure measurements), and laboratory tests (e.g., blood and urine biomarker analyses).

### 2.2. Study Population

Inclusion criteria were as follows: (1) adults (≥ 18 years old) without a history of diabetes or newly diagnosed patients with Type 2 diabetes (meeting the ADA criteria) and (2) data on predictive variables such as BMI, blood lipids (total cholesterol and high‐density lipoprotein [HDL]), smoking history, and physical activity duration.

Exclusion criteria were as follows: (1) excluding those with missing key predictive variables (e.g., BMI and family history), patients with secondary diabetes (e.g., caused by pancreatitis) or gestational diabetes, and those taking glucocorticoids (which affect blood glucose) and (2) those with “diabetes‐related records” (i.e., fasting blood glucose [FBG] ≥ 7.0 mmol/L, glycated hemoglobin [HbA1c] ≥ 6.5*%*, or with a doctor′s diagnosis record).

### 2.3. Predictor Variables

The primary outcome variable was participants′ diabetes status, which was categorized into nondiabetic, prediabetic, and diabetic. Diabetes was defined as (1) FBG ≥ 7.0 mmol/L (laboratory‐measured values), (2) self‐reported physician‐diagnosed diabetes with ongoing treatment (household questionnaire data), or (3) HbA1c ≥ 6.5*%*. The specific datasets included household questionnaire data, physical examination data, and laboratory data, as detailed in Table 1.

**Table 1:** Predictor variables.

Health ecology level	Dimension	Factors
Individual characteristic	Demographic characteristics	Age, gender, height, weight, and race
	Medical history	Arthritis; coronary heart disease; stroke; thyroid problem; chronic obstructive pulmonary disease (COPD), emphysema, or Chronic Hepatitis B (Chb); liver condition; cancer or malignancy; weak or failing kidneys; and high blood pressure
	Biological indicators	Total cholesterol, high‐density lipoprotein (HDL), lymphocyte percentage, monocyte percentage, segmented neutrophil percentage, eosinophil percentage, red blood cell (RBC) count, hemoglobin, RBC folate, Hepatitis A antibody, Hepatitis B surface antibody, C‐reactive protein, blood lead, blood cadmium, serum total folate, 17*Α*‐hydroxy pregnenolone, androstenedione, anti‐Mullerian hormone, and 25OHD2+25OHD3
Individual behavioral characteristics	Behavior	Smoke and sleep hours
	Diet	Coffee or tea with cream or sugar; alcohol; gum, mints, lozenges, or cough drops; and antacids, laxatives, or antidiarrheal
	Exercise	Minutes of sedentary activity and minutes of moderate‐intensity activity
	Mental health	Have little interest in doing things, feeling down, depression, or hopeless
Interpersonal relationship	Family relationships	Married and the total number of people in the household
	Societal relationship	Education
Living and working conditions	Working condition	Army force and occupation
	Living condition	Family income
	Medical conditions	general health condition and a routine place to go for health
Macroeconomic policy	Insurance	Covered by health insurance

### 2.4. Statistical Analysis

#### 2.4.1. Data Preprocessing

PyCharm 2023.1 (JetBrains s.r.o., Prague, Czech Republic) was adopted as the code development environment, with Python 3.9.13 as the underlying runtime environment. Specifically, pandas 2.0.3 was utilized for data management and missing value assessment, numpy 1.24.3 for numerical computation and imputation implementation, and scikit‐learn 1.2.2 for categorical variable encoding, correlation analysis, principal component analysis (PCA) dimensionality reduction, and standardization.

The workflow comprised the following steps: First, the dataset was systematically assessed for missing values, and categorical variables were appropriately coded. Second, variables or cases with missing values exceeding 30% were excluded from the analysis. For data with missing values below 30%, Monte Carlo multiple imputation was applied to handle the gaps. Subsequently, collinearity was evaluated using a correlation coefficient matrix; pairs of features exhibiting strong correlation (|*r*| > 0.8) were further analyzed using PCA to reduce dimensionality. Finally, all variables were standardized to normalize scales across features. *Z*‐score standardization is adopted to convert biochemical indicators (e.g., blood glucose and cholesterol) into a distribution with a mean of 0 and a standard deviation of 1, so as to avoid the impact of dimensional differences on the weight calculation of SVM and neural networks.

#### 2.4.2. Dataset Splitting

In this study, the dataset was split into 70% train set and 30% validation set for model development and evaluation.

#### 2.4.3. Data Analysis of Population Characteristics

Statistical analysis of the data in this study was performed using SPSS 22.0 (IBM Corporation, Armonk, New York, United States). For measurement data, if they followed a normal distribution, they were expressed as mean ± standard deviation, and the *t*‐test was used for comparison between groups; if they did not follow a normal distribution, they were expressed as median and interquartile range [*M* (P25, P75)]. Enumeration data were described using frequencies and percentages. Among the influencing factors, for measurement data that satisfied a normal distribution, the independent samples *t*‐test was adopted; for those with a nonnormal distribution, the Mann–Whitney *U* rank sum test was used. For enumeration data, the chi‐square (*χ* ^2^) test was applied. Since all data in this study are enumeration data, frequencies and percentages were used for statistical description, and the *χ* ^2^ test was employed for statistical analysis.

#### 2.4.4. Feature Selection

Model‐based feature selection methods used were RF and RFE. RF is an ensemble learning–based classification/regression algorithm that constructs multiple decision trees (DTs) and aggregates their results, simultaneously providing feature importance scores based on each feature′s contribution to predictive performance. RFE, an iterative feature selection method, operates on the principle of iteratively removing the least important features while retaining the subset of features with the most significant impact on model performance. Additionally, root mean square error (RMSE) was adopted as the criterion to determine the optimal number of features for selection. Based on this metric, the two feature selection methods were systematically compared to identify the optimal approach for feature selection.

#### 2.4.5. Imbalanced Data Handling

To address the unique challenge of the Pima dataset, namely, the class imbalance issue, this study applied multiple resampling techniques, including undersampling, oversampling, ADASYN, synthetic minority oversampling technique (SMOTE), and SMOTE combined with edited nearest neighbors (SMOTE‐ENN), to address class imbalance in the dataset. Following a systematic comparison, the optimal oversampling approach was selected for data preprocessing prior to model construction.

#### 2.4.6. Model Construction

Given that ensemble algorithms outperform single models in handling nonlinear relationships and high‐dimensional data, this study employed a series of machine learning models to analyze the data, each with distinct methodologies and advantages:

DT, a tree‐structured predictive model, leverages statistical probability to map object attributes to outcomes through hierarchical feature splits, with internal nodes representing attribute tests, branches denoting decision paths, and leaf nodes providing final predictions [18]. RF, an ensemble of independent DTs based on Bagging, enhances prediction accuracy and robustness by aggregating results from multiple trees via majority voting, demonstrating effectiveness in handling missing data and widely applied in disease risk prediction [19] and which is included for comparison because it can effectively handle interactions between features (e.g., the synergistic effect of BMI and dietary habits) and is less prone to overfitting. XGBoost iteratively adds weak DT learners to minimize loss functions, refining model performance by addressing residuals from prior iterations [20]. AdaBoost dynamically adjusts sample weights across iterations to combine weak classifiers into a strong one, improving classification accuracy through adaptive learning. The SVM aims to maximize classification margins by identifying optimal hyperplanes in high‐dimensional spaces, suitable for both classification and regression tasks [21]. In the sample characteristics of this study, there exist clearly linearly separable clinical indicators (e.g., blood glucose level and disease risk), and the selection of the linear kernel function for SVM offers significant advantages. The naive Bayesian model (NBM) is selected due to its significant advantage in computational efficiency for high‐dimensional small‐sample data and its insensitivity to missing values, which is consistent with the distribution characteristics of some features in this study [22]. Multilayer perceptron (MLP), a feedforward neural network with input, hidden, and output layers, employs nonlinear activation functions to tackle complex patterns, serving as a foundational model in artificial neural networks. These models, spanning tree‐based ensemble methods, statistical learning, and neural networks, were systematically evaluated to identify the optimal approach for the study′s predictive tasks.

#### 2.4.7. Modeling Assessment

The machine learning models were systematically evaluated using several key metrics: accuracy, precision, *F*1 score, recall, the receiver operating characteristic (ROC) curve, and the area under the curve (AUC). Accuracy, precision, *F*1 score, and recall were calculated based on the confusion matrix, which summarizes the model′s prediction performance across true positives, true negatives, false positives, and false negatives. The ROC curve illustrates the trade‐off between the true‐positive rate and false‐positive rate across classification thresholds, while the AUC quantifies the overall discriminative ability of the model, with values closer to 1 indicating superior performance. These metrics collectively provide a comprehensive assessment of the models′ predictive accuracy, reliability, and generalizability.

#### 2.4.8. Model Validation

Model validation primarily utilized *k*‐fold cross‐validation. The detailed workflow of this method is as follows: First, the complete dataset was precisely and uniformly partitioned into *k* subsets of comparable size. In each validation iteration, one subset was carefully designated as the test set to evaluate model performance, while the remaining *k*‐1 subsets were combined as the training set for model training. This process was repeated iteratively *k* times to comprehensively assess the model′s effectiveness and stability. During each validation cycle, key evaluation metrics such as sensitivity, specificity, and AUC were recorded. After completing all *k* iterations, the mean values of these metrics were calculated to serve as the final evaluation basis. When *k* is set to 5 or 10, this approach achieves an optimal balance between evaluation accuracy and computational complexity. By implementing this method, the performance of different learning algorithms can be accurately assessed and effectively compared, providing reliable data support for selecting the most suitable algorithm.

#### 2.4.9. Model Hyperparametric Tuning

Hyperparameter tuning represents a pivotal step in machine learning and deep learning methodologies, aiming to optimize model performance through the adjustment of external configuration parameters [24]. This study primarily employed random search for hyperparameter optimization.

## 3. Results

### 3.1. Population Characteristics

**Figure 1:**
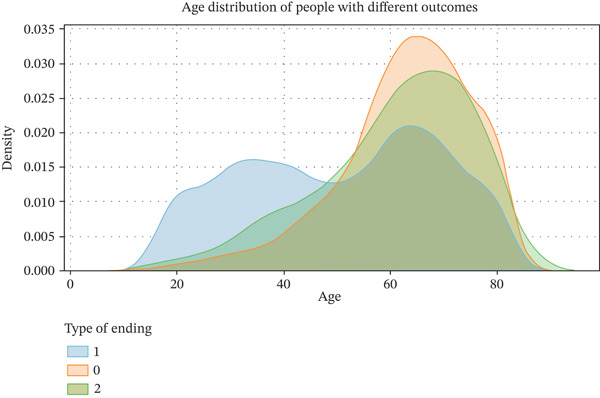
Age distribution of populations with different T2DM statuses (years).

The database initially comprised 11,933 study participants, among whom 3073 lacked laboratory examination data and 2523 were younger than 18 years old. After applying inclusion and exclusion criteria, a total of 6337 participants were ultimately enrolled in the study. The mean age of the study subjects was 52.33 ± 18.28 years, with 3479 females (54.8%) and 2858 males (45.1%). There are statistical differences in key characteristics such as age, comorbidities (e.g., hypertension, kidney disease, and heart disease), and medical insurance between the diabetic and nondiabetic populations. Age distribution by T2DM status is shown in Figure 1, and the baseline of participants is shown in Table 2.

**Table 2:** Baseline characteristics of the study participants (mean ± standard deviation/frequency, %).

Variables	Total (*n* = 6337)	Non‐T2DM (*n* = 871)	Prediabetes (*n* = 5243)	T2DM (*n* = 223)	Statistic	*p*
Age (years)	52.33 ± 18.28	63.18 ± 12.33	50.16 ± 18.51	60.86 ± 14.35	*F* = 230.16	< 0.001
Weight (g)	147.23 ± 889.26	177.73 ± 1037.56	139.93 ± 849.19	199.78 ± 1146.88	*F* = 1.08	0.340
Height (cm)	164.98 ± 20.64	162.96 ± 24.69	165.38 ± 19.82	163.37 ± 21.64	*F* = 5.81	0.003
Total cholesterol (mg/dL)	186.97 ± 42.67	168.86 ± 44.97	190.04 ± 41.52	184.42 ± 42.91	*F* = 84.75	< 0.001
High‐density lipoprotein (mg/dL)	54.41 ± 14.69	48.64 ± 12.80	55.43 ± 14.82	52.46 ± 12.99	*F* = 74.76	< 0.001
Gender, *n* (%)					*χ* ^2^ = 6.92	0.031
Male	2858 (45.10)	427 (49.02)	2326 (44.36)	105 (47.09)		
Female	3479 (54.90)	444 (50.98)	2917 (55.64)	118 (52.91)		
Race, *n* (%)					*χ* ^2^ = 6.426	0.040
Mexican American	448 (7.07)	70 (8.04)	363 (6.92)	15 (6.73)		
Other Hispanic	647 (10.21)	93 (10.68)	529 (10.09)	25 (11.21)		
Non‐Hispanic White	3684 (58.13)	443 (50.86)	3125 (59.60)	116 (52.02)		
Non‐Hispanic Black	791 (12.48)	149 (17.11)	614 (11.71)	28 (12.56)		
Non‐Hispanic Asian	353 (5.57)	43 (4.94)	282 (5.38)	28 (12.56)		
Other race	414 (6.53)	73 (8.38)	330 (6.29)	11 (4.93)		
Education, *n* (%)					*χ* ^2^ = 162.628	< 0.001
Less than 9^th^ grade	292 (4.82)	78 (8.99)	193 (3.88)	21 (9.50)		
9–11^th^ grade	482 (7.95)	120 (13.82)	337 (6.77)	25 (11.31)		
High school graduate/GED or equivalent	1297 (21.39)	224 (25.81)	1024 (20.58)	49 (22.17)		
Some college or AA degree	1846 (30.44)	270 (31.11)	1513 (30.41)	63 (28.51)		
College graduate or above	2143 (35.34)	174 (20.05)	1906 (38.31)	63 (28.51)		
Marital status, *n* (%)					*χ* ^2^ = 1.982	0.371
Married/living with partner	3277 (54.05)	422 (48.62)	2729 (54.87)	126 (57.01)		
Widowed/divorced/separated	1536 (25.33)	326 (37.56)	1151 (23.14)	59 (26.70)		
Never married	1246 (20.55)	120 (13.82)	1090 (21.91)	36 (16.29)		
Smoke, *n* (%)					*χ* ^2^ = 61.073	< 0.001
Yes	2518 (39.75)	438 (50.29)	1968 (37.55)	112 (50.22)		
No	3810 (60.14)	432 (49.60)	3267 (62.34)	111 (49.78)		
Occupation, *n* (%)					*χ* ^2^ = 236.136	< 0.001
Working at a job or business	3229 (50.95)	262 (30.08)	2881 (54.95)	86 (38.57)		
With a job or business but not at work	203 (3.20)	19 (2.18)	180 (3.43)	4 (1.79)		
Looking for work	264 (4.17)	26 (2.99)	226 (4.31)	12 (5.38)		
Not working at a job	2640 (41.66)	564 (64.75)	1955 (37.29)	121 (54.26)		
Heart disease, *n* (%)					*χ* ^2^ = 117.854	< 0.001
Yes	322 (5.31)	119 (13.71)	184 (3.70)	19 (8.60)		
No	5716 (94.28)	739 (85.14)	4776 (96.02)	201 (90.95)		
Stroke, *n* (%)					*χ* ^2^ = 75.062	< 0.001
Yes	280 (4.62)	92 (10.60)	174 (3.50)	14 (6.33)		
No	5773 (95.22)	772 (88.94)	4794 (96.38)	207 (93.67)		
Weak/failing kidney, *n* (%)					*χ* ^2^ = 170.795	< 0.001
Yes	231 (3.81)	102 (11.75)	127 (2.55)	2 (0.90)		
No	5822 (96.01)	765 (88.13)	4839 (97.27)	218 (98.64)		
Covered by health insurance, *n* (%)					*χ* ^2^ = 22.935	< 0.001
Yes	5794 (91.43)	831 (95.41)	4754 (90.67)	209 (93.72)		
No	529 (8.35)	39 (4.48)	476 (9.08)	14 (6.28)		
Hypertension, *n* (%)					*χ* ^2^ = 623.975	< 0.001
Yes	2323 (36.66)	626 (71.87)	1562 (29.79)	135 (60.54)		
No	4010 (63.28)	244 (28.01)	3678 (70.15)	88 (39.46)		

Abbreviations: *χ* ^2^, chi‐square test; *F*, ANOVA.

### 3.2. Colinearity Check

Collinearity checks were performed for the variables, revealing strong pairwise correlations: stroke and coronary heart disease (correlation coefficient = 0.88), weak or failing kidneys and stroke (correlation coefficient = 0.84), and weak or failing kidneys and coronary heart disease (correlation coefficient = 0.83). PCA extracted two principal components that collectively explained 96.12% of the variance.

### 3.3. Feature Selection

Two model‐based feature selection methods (RF and RFE) were employed to reduce data dimensionality. RF quantified feature importance and selected 24 features with scores above the mean using RMSE as the criterion, retaining core variables with significant impacts on the outcome. RFE iteratively eliminated less important features and identified an optimal set of five features via cross‐validation, prioritizing strict dimensionality reduction to control model complexity.

**Figure 2:**
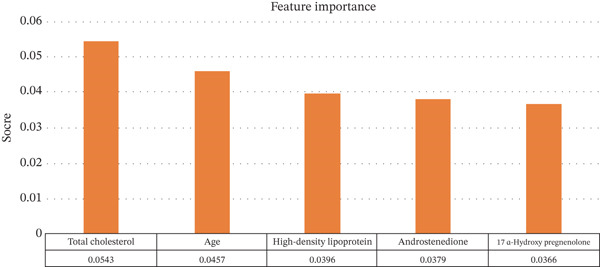
Score ranking of partial high‐importance features in the RF‐selected T2DM prediction model.

**Figure 3:**
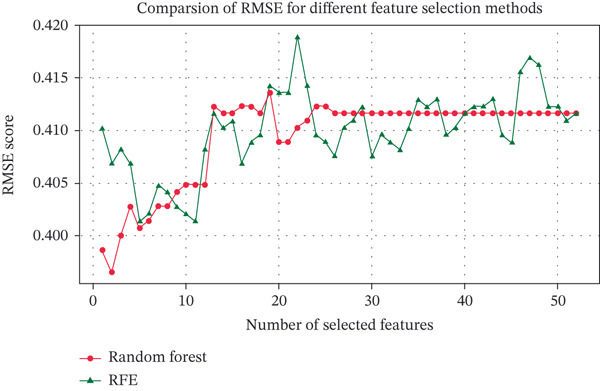
Comparison of RMSE for different feature selection methods.

Comparative analysis showed that RF achieved lower prediction error (minimum RMSE = 0.3966), balancing model complexity and predictive accuracy with its feature subset, whereas RFE exhibited slightly higher RMSE (minimum RMSE = 0.4014) when limiting feature quantity. Integrating methodological principles and performance evaluation, RF emerged as the optimal choice due to its capability to capture nonlinear relationships and robust feature ranking (refer to Figure 2). The 24‐dimensional feature subset selected by RF provided an input variable set with both interpretability and predictive efficacy for subsequent model development, including the following: height, weight, HDL, total cholesterol, hypertension, advanced glycation end products (AGEs), testosterone, 17*α*‐hydroxyprogesterone, antiovarian hormones, blood lead, 25‐hydroxyvitamin D, blood cadmium, hemoglobin, red blood cells, C‐reactive protein, lymphocytes, segmented neutrophils, folate, total serum folate, sleep duration, physical activity duration, sedentary time, income, and medical conditions. To visually present the quantification results of feature importance by RF, Figure 3 displays the score distribution of some high‐importance indicators among the 24 core features selected in this study (the higher the score, the greater the contribution of the corresponding feature to the T2DM status prediction model). The results show that the ranking of the top five features in terms of contribution is as follows: total cholesterol > age > HDL > androstenedione > 17*α*‐hydroxy pregnenolone, and these are the core input variables that play a key role in the outcome variable during subsequent model construction.

### 3.4. Imbalance Data Handling

Table 3 presents the performance of machine learning algorithms with different data balancing techniques. ADASYN consistently outperformed all other methods in terms of accuracy, demonstrating superior data balancing effectiveness in this study.

**Table 3:** Comparison of imbalanced data handling techniques across machine learning algorithms.

Algorithms	Performance metrics	Unbalanced data	Undersampling	Oversampling	ADASYN	SMOTE	SMOTE‐ENN
DT	Accuracy	0.75	0.40	0.37	0.60	0.59	0.52
	AUC	0.59	0.55	0.53	0.71	0.70	0.65
RF	Accuracy	0.85	0.55	0.40	0.62	0.61	0.56
	AUC	0.77	0.72	0.71	0.85	0.86	0.78
XGBoost	Accuracy	0.85	0.49	0.45	0.68	0.73	0.60
	AUC	0.76	0.68	0.71	0.86	0.68	0.80
AdaBoost	Accuracy	0.84	0.48	0.54	0.71	0.86	0.63
	AUC	0.70	0.65	0.66	0.85	0.87	0.79
SVM	Accuracy	0.85	0.51	0.48	0.53	0.53	0.60
	AUC	0.75	0.71	0.67	0.76	0.76	0.76
NBM	Accuracy	0.72	0.29	0.36	0.39	0.40	0.50
	AUC	0.72	0.64	0.60	0.63	0.65	0.74
MLP	Accuracy	0.84	0.49	0.44	0.52	0.50	0.55
	AUC	0.77	0.67	0.66	0.74	0.74	0.75

### 3.5. Model Construction

**Figure 4:**
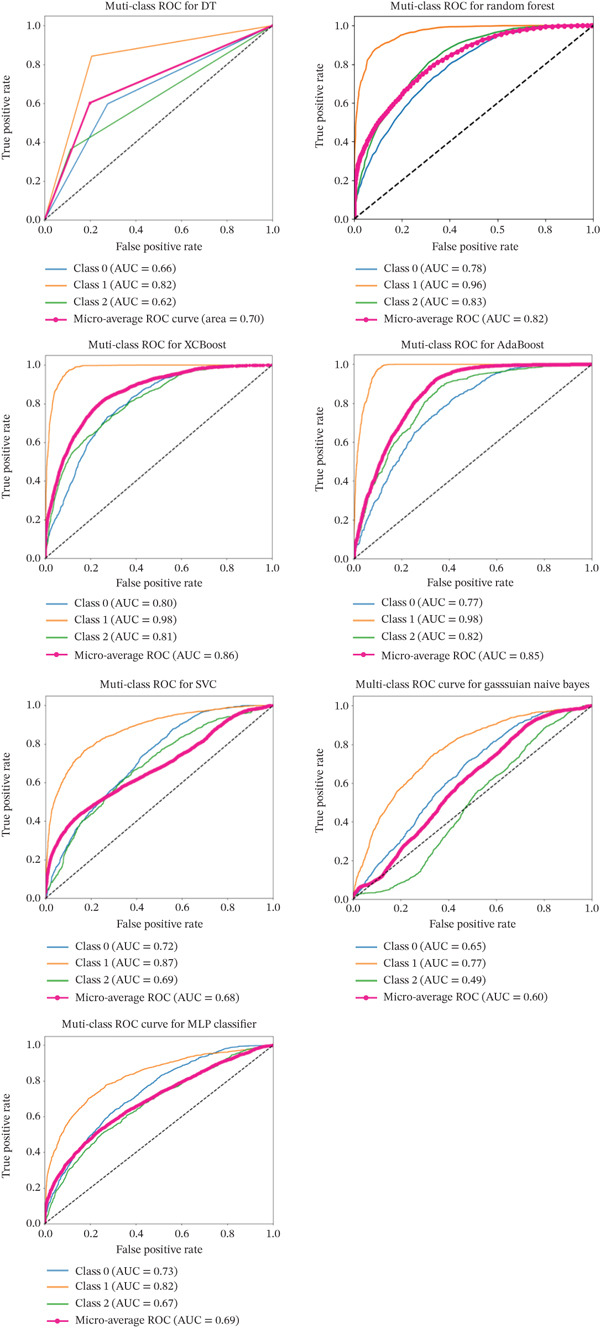
ROC curves comparing the performance of different machine learning models.

ADASYN was applied for oversampling and subsequent model development. Performance metrics for each model are presented in Table 4, and the ROC curves are shown in Figure 4. Among the models, XGBoost achieved the highest performance, with an AUC of 0.86 (95% CI: 0.85, 0.87), an accuracy of 0.68 (95% CI: 0.67, 0.69), and a precision of 0.72 (95% CI: 0.71, 0.73).

**Table 4:** Performance comparison of different models (95% confidence interval).

Algorithms	AUC	Accuracy	Precision	Sensitivity	*F*1
DT	0.70 (0.69, 0.71)	0.60 (0.59, 0.62)	0.60 (0.59, 0.62)	0.60 (0.59, 0.62)	0.59 (0.57, 0.60)
RF	0.85 (0.85, 0.86)	0.62 (0.61, 0.63)	0.67 (0.66, 0.69)	0.62 (0.61, 0.63)	0.56 (0.55, 0.58)
XGBoost	0.86 (0.85, 0.87)	0.68 (0.67, 0.69)	0.72 (0.71, 0.73)	0.68 (0.67, 0.69)	0.65 (0.64, 0.66)
AdaBoost	0.85 (0.85, 0.86)	0.71 (0.70, 0.72)	0.71 (0.70, 0.73)	0.71 (0.70, 0.72)	0.71 (0.70, 0.72)
SVM	0.76 (0.75, 0.77)	0.53 (0.52, 0.55)	0.55 (0.53, 0.57)	0.53 (0.52, 0.54)	0.47 (0.46, 0.49)
NBM	0.63 (0.62, 0.64)	0.39 (0.38, 0.40)	0.51 (0.49, 0.53)	0.39 (0.38, 0.40)	0.35 (0.34, 0.36)
MLP	0.74 (0.73, 0.75)	0.52 (0.51, 0.54)	0.56 (0.54, 0.57)	0.52 (0.51, 0.53)	0.48 (0.46, 0.49)

Internal validation using 10‐fold cross‐validation on the validation set yielded an accuracy of 0.95 (95% CI: 0.949–0.954) and precision of 0.95 (95% CI: 0.948–0.954). Random search was employed to optimize model parameters, resulting in improved performance for AdaBoost. After optimization, the model achieved an accuracy of 0.903 ± 0.0114 and an AUC of 0.97 ± 0.004, reflecting enhanced predictive efficiency.

## 4. Discussion

### 4.1. Analysis of Risk Factors for the Development of T2DM Based on the HEM

In this study, 24 risk factors predicting the development of T2DM were identified by machine learning algorithms and analyzed hierarchically according to the HEM. This approach is aimed at providing multilevel entry points for future T2DM prevention and intervention. The HEM integrates concepts from medicine, psychology, sociology, and environmental science [25]. It examines not only the impact of individual diseases on health but also explores the impact of three upstream layers, namely, interpersonal networks, work and living conditions, and macropolicies, on health outcomes, with individual traits, behaviors, and lifestyles as the core layer [26].

#### 4.1.1. Analysis of Risk Factors for the Development of T2DM Based on the Individual Trait Level

Studies have found that indicators such as height, weight, HDL, total cholesterol, history of hypertension, AGEs, testosterone, 17*α*‐hydroxyprogesterone, antiovarian hormones, blood lead, 25‐hydroxyvitamin D, blood cadmium, hemoglobin, red blood cells, C‐reactive protein, lymphocytes, segmented neutrophils, folate, and total serum folate are closely associated with abnormal insulin secretion and insulin resistance, consistent with previous research findings [27]. Among these, height and weight indirectly correlate with insulin resistance by affecting BMI and fat distribution [28]; dyslipidemia (decreased HDL and elevated total cholesterol), hypertension (which impairs vascular endothelial function), and AGEs (which activate oxidative stress and inflammation) can all induce or exacerbate insulin resistance [29–33], suggesting that individuals should pay attention to monitoring weight, blood pressure, and blood lipids, and improve unhealthy living habits. Elevated lymphocyte and neutrophil counts indicate an inflammatory response in the body [34, 35]; abnormal levels of hormones such as testosterone and 17*α*‐hydroxyprogesterone reflect endocrine disorders [36–38]; lead and cadmium poisoning, as well as 25‐hydroxyvitamin D deficiency, can impair insulin secretion and receptor function [39, 40]; abnormal hemoglobin and red blood cell levels are associated with nutritional status and chronic diseases, which in turn affect glucose metabolism [41]; folate deficiency can increase homocysteine levels and elevate the risk of disease [42], highlighting the preventive value of nutritional factors.

In summary, the aforementioned biochemical indicators can reflect the status of multiple systems in the body, such as immunity, endocrine function, and metabolism, and provide important biological evidence for the early identification, mechanism research, and clinical intervention of T2DM.

#### 4.1.2. Analysis of Risk Factors for the Development of T2DM Based on Individual Behavioral Level

At the individual behavioral level, this study found that sleep duration, physical activity duration, and sedentary time were risk factors for the development of T2DM, which is to some extent related to the accelerated pace of modern life and increased work stress. Lack of sleep not only increases the level of high‐sensitivity C‐reactive protein, which mediates inflammation, but also heightens the excitability of the sympathetic nervous system and cortisol secretion, which promotes gluconeogenesis [43]. Short‐term sleep increases the body′s appetite, leading to excess energy intake and weight gain [44]. Both short periods of physical activity and sedentary time lead to low energy expenditure, reduced muscle uptake and utilization of glucose, slowed fat metabolism, and increased concentrations of circulating proinflammatory mediators, which in turn exacerbate insulin resistance [45, 46], highlighting the importance of both rest and activity for metabolic health.

#### 4.1.3. Risk Analysis of the Occurrence of T2DM Based on the Dimension of Work and Living Conditions

At the level of working and living conditions, this study found that income and medical conditions were risk factors for the development of diabetes, reflecting the impact of differences in economic levels and medical resources. Low‐income populations may lack the resources needed for physical activity and health knowledge related to diabetes prevention [47], and populations with poor medical care may lack regular screening and long‐term management, leading to missed opportunities for early T2DM prevention and treatment [48]. This suggests a need for improved T2DM health education and expanded healthcare infrastructure to enhance screening and management accessibility.

This multilevel risk factor analysis, based on the health ecology perspective, not only addresses the limitation of focusing solely on individual factors but also provides a researchable direction for future T2DM health management research.

### 4.2. Analysis of Machine Learning–Based Prediction Model Construction for T2DM Occurrence

Existing studies have mostly explored the problem of predicting the risk of disease occurrence with traditional regression analysis, but traditional regression methods often suffer from limitations such as small sample sizes, inability to deal with nonlinear relationships, restricted data types, and unbalanced samples, resulting in limited model prediction accuracy and insufficient generalization ability [49]. A comparison with the traditional clinical risk score FINDRISC reveals that the AUC value of this study is higher (0.85 vs. 0.72), which may be attributed to the inclusion of more biochemical indicators (e.g., blood lead and folic acid) and social factors, thereby improving the prediction accuracy [50]. With the advent of the big data era, machine learning algorithms have demonstrated powerful large‐scale data processing capabilities and have gradually been applied as modern medical information technology in the field of disease occurrence and prognosis assessment [50, 51]. Olivera et al. developed and validated a prediction model for detecting undiagnosed diabetes using data from the Brazilian Longitudinal Study of Adult Health (ELSA‐Brasil) and compared the performance of different machine learning algorithms in this task, without any handling of class imbalance [52]. This study compares different methods for handling unbalanced datasets, and adaptive synthetic sampling significantly outperforms the other methods in terms of accuracy. The method ADASYN is aimed at avoiding overamplification of noise or anomalies in a few classes, thus reducing the possibility of overfitting, and better highlights samples from a few classes near the classification boundaries to help the classifier learn more accurate decision boundaries, which makes the model more adaptive and predictive when facing all types of data in practical applications [53]. In this study, we found that the AdaBoost model demonstrated higher precision, sensitivity, and *F*1 score than other models, with AUC and accuracy comparable to those of the XGBoost model. The closer the AUC value is to 1, the better the classification performance of the model and the better the identification ability, indicating that the model can accurately identify high‐risk diabetic patients; the closer the *F*1 score is to 1, the better the precision and robustness of the model, indicating that the model precision rate and recall are more balanced, which can reduce the missed diagnosis of high‐risk patients and misdiagnosis of low‐risk patients [52]. In comparison, the AdaBoost model showed the best performance in predicting the risk of developing T2DM. This is an iterative‐based integrated learning algorithm that builds a strong learner by combining multiple weak learners (e.g., simple models, e.g., DT stumps), is able to improve the overall accuracy by continuously adjusting the sample weights so that the classifier can better learn the sample features [54], and is now being progressively applied to diseases related to the endocrine system [55]. Song et al. [56] used AdaBoost for the classification of glucose‐lowering drug regimens for T2DM patients, and the algorithm demonstrated effectiveness in the classification task.

In summary, the best AdaBoost model combines easy‐to‐get indicators and can be used for rapid diabetes risk screening. This model can be integrated into the electronic health systems of primary care settings. By inputting easily accessible indicators such as height, weight, and blood pressure, it rapidly generates a diabetes risk score (0–100 points), and individuals with a score ≥ 60 are recommended to undergo further laboratory tests, making it suitable for community doctors and public health personnel to screen high‐risk groups. The multidimensional risk analysis based on the health ecology framework identifies relevant factors in social behavior scenarios. It provides a basis for formulating group intervention strategies, such as “workplace health promotion,” and also offers references for policies like medical resource allocation and medical insurance coverage. Modifiable risk factors for patients and high‐risk groups point out a clear direction for self‐management. In short, this study combines individual behaviors, clinical practice, and social policies, providing an operable plan for precise diabetes prevention and control and the realization of health equity.

## 5. Conclusion

This study identified key risk factors and constructed a machine learning prediction model for T2DM, guided by the health ecology framework. Theoretically, by constructing a machine learning prediction model based on multidimensional biochemical indicators (e.g., immune, endocrine, and metabolic markers), this study reveals the potential synergistic risk mechanisms underlying the pathogenesis of Type 2 diabetes, thereby providing a new perspective for etiological research on the disease. Practically, the model′s efficacy in identifying high‐risk populations is superior to that of traditional clinical scoring systems. It can serve as a clinical decision support tool for early screening and precise intervention in primary healthcare, helping to reduce the disease burden.

## 6. Limitation

This study has several important limitations that should be acknowledged. First, the use of retrospective data introduces inherent challenges, such as potential biases from incomplete or inaccurately recorded historical variables, which may affect the validity of associations identified. While missing data were handled using established methods such as multiple imputation, residual uncertainty in the imputed values may affect the robustness of the result. Additionally, the absence of external validation with independent datasets limits the generalizability of the findings, as the model performance and feature importance identified herein may not be reproducible in populations with different demographic, clinical, or environmental characteristics.

## 7. Future Perspectives

Future research can be advanced in four aspects: first, conducting prospective cohort studies to clarify the temporal association between risk factors and the onset of Type 2 diabetes, thereby strengthening causal evidence; second, performing external validation in populations of different ethnicities and geographical regions to optimize the generalizability of the model; third, integrating longitudinal exposure data such as continuous glucose monitoring and environmental pollutant tracking to enhance the timeliness and accuracy of the model; and fourth, developing mobile applications to achieve real‐time risk early warning and public self‐assessment, thereby facilitating the deployment of the model in real‐world medical settings.

## Author Contributions

Qian Xu, Ruicong Yu, and Jing Sun substantially contributed to conception and design, acquisition of data, and analysis and interpretation of data. Qian Xu, Ruicong Yu, Yannan Jiang, and Huixin Qiu involved in drafting the manuscript or revising it critically for important intellectual content and drafting the manuscript. Cuirong Xu and Jing Sun critically reviewed and edited the manuscript and agreed to be accountable for all aspects of the work in ensuring that questions related to the accuracy or integrity of any part of the work are appropriately investigated and resolved. Qian Xu and Ruicong Yu are co‐first authors.

## Funding

This study was supported by the Jiangsu Commission of Health, 10.13039/100017962, ZD2022057.

## Conflicts of Interest

The authors declare no conflicts of interest.

## Data Availability

The data that support the findings of this study are openly available in the National Health and Nutrition Examination Survey at https://wwwn.cdc.gov/nchs/nhanes/Default.aspx.
